# Tackling Cravings in Medical Weight Management: An Update on Pathophysiology and an Integrated Approach to Treatment

**DOI:** 10.3390/nu16193238

**Published:** 2024-09-25

**Authors:** Naomi Kakoschke, Belinda A. Henry, Michael A. Cowley, Kevin Lee

**Affiliations:** 1 Health & Biosecurity, Commonwealth Scientific Industrial Research Organisation (CSIRO), Adelaide 5000, Australia; 2 Faculty of Medicine, Nursing and Health Sciences, Monash University, Melbourne 3800, Australia; belinda.henry@monash.edu (B.A.H.); michael.cowley@monash.edu (M.A.C.); kevin.lee@monash.edu (K.L.); 3 Parkside Specialists, Melbourne 3004, Australia

**Keywords:** cravings, mindfulness, CBT, Contrave, Semaglutide, Tirzepatide

## Abstract

**Background/Objectives**: Food cravings involve a strong drive to consume palatable foods irrespective of nutritional status. Importantly, cravings contribute substantially to the obesity epidemic. Managing hunger alone is insufficient for weight management as this relates only to homeostatic eating and does not address the complex aetiology of hedonic eating and its crucial role in food cravings. Medical weight management clinics and anti-obesity medication trials do not routinely identify and address food cravings. **Methods**: We conducted a narrative review of the literature consisting of 115 peer-reviewed articles (original articles and reviews). We included articles focused on food craving pathophysiology, assessment, and management strategies providing contrasts against the current medical model of weight management seen in obesity pharmacotherapy trials as well as the current standard of practise. **Results**: We outline the neurohormonal and psychological drivers of cravings, which lead to a spectrum of eating behaviours, from comfort food eating to binge eating disorders. We provide an overview of ways of identification and measurement options, including their strengths and weaknesses, and an overview of management strategies and their cravings control efficacy, spanning lifestyle modifications like nutrition and sleep, psychological therapies (i.e., cognitive behavioural therapy [CBT], acceptance-based therapies such as mindfulness) and, last but not least, medications that not only are approved for weight reduction but reduce cravings. Finally, based on these findings, we provide a proposed integrated and iterative model that is able to evolve and adapt to the individual over time in tackling cravings for long-term weight loss maintenance. **Conclusions**: The findings emphasise the importance of cravings management and provide a synthesis on how cravings can be identified in a medical weight management setting, which can be practically implemented in an integrated iterative model spanning anti-obesity medications that have craving control data to evidence-based lifestyle and psychological interventions.

## 1. Introduction

Hypocaloric dieting in combination with anti-obesity medications is currently the mainstay of medical weight management, but this approach does not take into account food cravings. Food cravings can be defined as a desire to eat a certain food in the absence of metabolic need (i.e., hunger). They are distinct from homeostatic eating or hunger when driven by the physiological need to meet energy demands and conversely are considered a part of hedonic eating, with compulsion to eat being driven through the desire to obtain pleasure in the absence of an energy deficit. Thus, whilst cravings can be considered a common occurrence amongst the general population, they can also be considered a marker of disordered eating patterns.

Generally, food cravings are divided into tonic and phasic cravings. Tonic cravings relate to spontaneously arising cravings measured retrospectively over a period, while phasic cravings relate to the provocation of cravings, for example, by food-related cues. Phasic cravings are known to be increased during the luteal phase of the menstrual cycle [1,2,3,4], during pregnancy, and in response to stress [5,6], indicating that they can be triggered by the hormonal milieu as well as emotional and external cues. Moreover, there appear to be sex differences in the experience of cravings, which are more prevalent in women than men, particularly during periods of stress [7,8] with some exceptions depending on type of stressor [9].

Several validated psychological constructs have been proposed to describe the phenomenon of non-hunger eating or eating in the absence of an energy deficit, including disinhibited eating, emotional eating, food addiction [10], reward-induced eating [11], and uncontrolled eating [12]. The term uncontrolled eating shows promise in encapsulating and unifying the spectrum or continuum of non-hunger or non-homeostatic eating patterns, from mild forms of uncontrolled eating such as comfort food eating and cravings manifest to severe forms including binge eating. There are, however, limitations to this construct as not all hedonic eating is uncontrolled and the term, whilst taking into account the continuum of loss of control in eating, lacks the nuance of describing the facet of that loss in terms of how that behaviour is taking place, be it food cue-related, habituated eating, or endocrine-driven with regard to stress or menstrual cyclicity [13,14,15].

Regardless of the construct used, it is known that cravings contribute substantially to the obesity epidemic [16]. Specifically, meta-analyses indicate that food cravings predict eating and weight gain, and that controlling food cravings are fundamental to the success of long-term weight loss maintenance [17]. Among individuals who are struggling to maintain weight loss, despite having had bariatric surgery or anti-obesity medication, cravings in the form of stress-related eating and binge eating episodes are known to negatively impact weight loss effectiveness and the strict control of eating is antecedent to binge eating episodes [18] and weight regain [19].

There are numerous theoretical models of cravings that span cognition, psychobiology, and cue conditioning components. One of the most influential theories is the Elaborated Intrusion (EI) Theory of Desire [20]. EI theory posits that cravings (desire) reflect a motivated state driven by two different cognitive processes: (1) an internal or external cue that automatically prompts an intrusive thought regarding an appealing target, and (2) elaboration on this thought, which maintains its salience and boosts motivations to obtain the target. In the context of palatable food, the initial process may involve an advertisement for a chocolate bar (i.e., an external cue) prompting a thought about eating one. The elaboration component may involve thinking about eating the chocolate bar in vivid detail from a sensory perspective, including imagining how it looks, tastes, and feels in addition to thinking about a previous consumption occurrence. While focused on the cognitive component, EI theory also integrates elements of cue conditioning as well as motivation.

This review examined the available treatment options for tackling stress and emotional forms of food craving, including lifestyle modification (e.g., macronutrient manipulation, sleep improvement, stress reduction), psychological therapies (e.g., CBT and acceptance-based approaches), and anti-obesity medications, with data supporting craving reduction or control. The objective of the review was to provide a practical guide that helps integrate these modalities to enhance the current standard of care in medical weight management clinics, making it holistic, person-centred, and adaptive to individual needs.

## 2. Method

This narrative review included 115 articles consisting of peer-reviewed publications (original research articles or reviews only) derived from a PubMed search conducted from July to September 2024 for articles dating from 2010 to 2024. Seminal papers illustrating important concepts were also included, but there were no publication date restrictions.

## 3. Pathophysiology of Cravings

The drive to eat can be attributed to three factors, namely, hunger, pleasure (or hedonic eating), and cognition (or executive function). These three factors co-exist in varying degrees, contributing to our eating choices and eating behaviours. It is important to highlight that when it comes to cravings, whether we eat or not is a function of these three factors. There is a lack of impulse control regarding cravings caused by insufficient input from executive function, and cravings, although they can exist independent of hunger, are exacerbated by homeostatic hunger, further impairing the ability to control them. Overcoming the lack of control is integral to mitigating the impact of cravings on weight management.

As described above, uncontrolled eating as a construct is becoming an accepted term that encapsulates the spectrum of problematic eating behaviour, from mild forms like comfort food eating, stress-related eating, and cravings to severe forms of the trait such as binge eating disorder [21]. Various studies have shown that uncontrolled eating correlates positively with weight gain over time [22]. Furthermore, uncontrolled eating is negatively correlated with successful weight loss and is associated with poor cognitive control, negative affective bias, and reduced reward sensitivity [23]. Cravings, though mild in the continuum of uncontrolled eating, are nonetheless significant in their impact on weight management and although not a disease per se are a physiological phenomenon that become problematic regarding unhealthy or undesired weight gain.

The neurobiological mechanisms that govern cravings are highly complex. Within the brain, there are distinct neural pathways regulating the homeostatic and non-homeostatic aspects of food intake. However, given that hunger exacerbates food cravings, it is not surprising that we see convergence and integration of the two neuronal systems [24,25].

**Figure 1:**
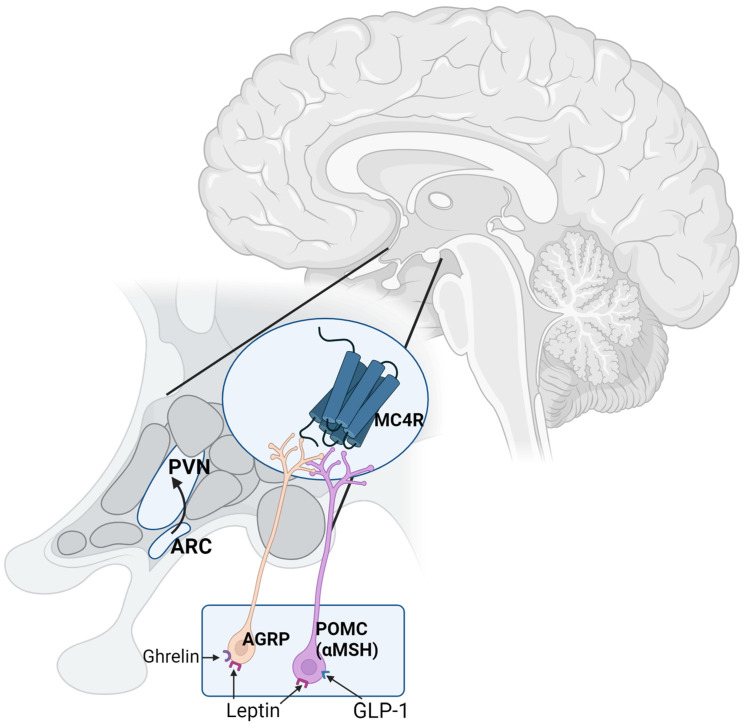
Homeostatic hunger is primarily controlled by the hypothalamus. Energy deficit is signalled to the brain, in particular the arcuate nucleus (ARC) of the hypothalamus, via various circulating factors, including the hormones ghrelin, leptin, and glucagon-like peptide 1 (GLP-1). Within the arcuate nucleus there are two key neuronal populations, including the proopiomelanocortin (POMC) neurons that signal satiety via release of alpha-melanocyte-stimulating hormone (alpha MSH) and the agouti-related protein (AGRP) neurons that promote hunger. Both alphaMSH and AGRP regulate food intake via action at the melanocortin 4 receptor (MC4R) located in the paraventricular nucleus (PVN) of the hypothalamus; aMSH activates MC4R to cause satiety and AGRP is the endogenous antagonist, blocking MC4R to increase food intake. Leptin regulates food intake by activating the POMC neuron and inhibiting the AGRP neuron; this contrasts the effect of GLP-1, which has a direct effect on POMC neurons only. Ghrelin increases food intake via activating the AGRP neurons. Homeostatic hunger is driven by an activation of the AGRP neurons and the inhibition of the POMC neurons (figure drawn by author).

Within the hypothalamus, the arcuate nucleus/tuberoinfundibular zone is integral to the homeostatic control of food intake in response to energy fluctuations as well as being an important regulator of cravings [24,25,26]. The arcuate nucleus contains two key distinct neuronal subpopulations, namely, satiety neurons that expression proopiomelanocortin (POMC) and the orexigenic neurons containing neuropeptide Y (NPY) and agouti-related protein (AGRP) (Figure 1). These neurons receive peripheral information regarding both acute and chronic nutritional states from various factors, including the adipocyte-derived hormone that promotes satiety called leptin and the hunger hormone ghrelin secreted from the stomach [26]. In times of caloric or energy deficit, POMC neurons are inhibited and the NPY/AGRP neurons are activated to promote homeostatic hunger pathways and to increase food intake. To do this, POMC neurons synthesise and secrete melanocyte-stimulating hormone (αMSH), which reduces food intake by acting at the melanocortin 4 receptor (MC4R); AGRP is the endogenous antagonist of αMSH at the MC4R and thus promotes hunger. This well-established pathway is a key target for the development of a new generation of weight loss pharmacotherapies, including GLP-1 agonists (discussed in detail below) and the MC4R agonist, setmelanotide [27], which is used to treat monogenic and syndromic forms of obesity.

**Figure 2:**
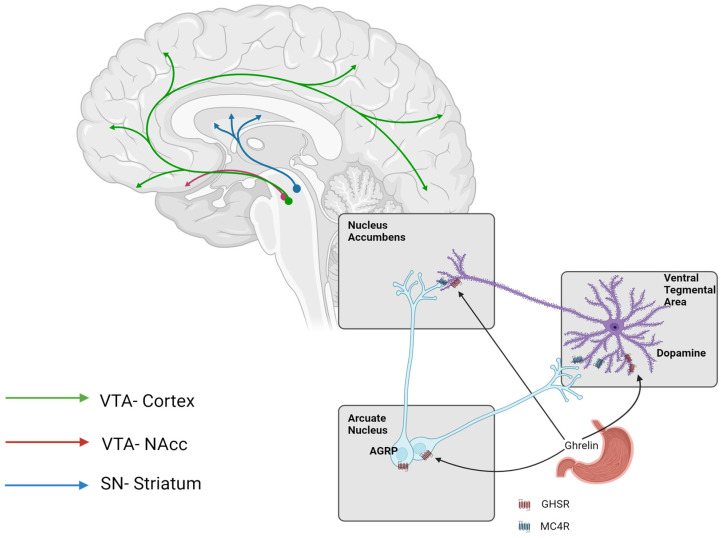
The mesolimbic dopamine reward pathway is central to the control of non-homeostatic control of food intake and craving-associated eating patterns. Dopaminergic neurons within the ventral tegmental area (VTA) provide input into both the prefrontal cortex and the nucleus accumbens (NAcc), whereas dopaminergic neurons within the substantia nigra (SN) project to the striatum brain region. Energy deficit, through increased secretion of ghrelin and increased activation of agouti-related protein (AGRP) neurons in the arcuate nucleus of the hypothalamus, not only increases homeostatic hunger but can also exacerbate cravings via the modulation of the dopamine system. Ghrelin can potentiate dopaminergic action via the growth hormone secretagogue receptors located in the NAcc and the VTA. In addition, AGRP increases dopaminergic signalling via the melanocortin 4 receptor (MC4R) located in the same brain regions. This figure illustrates the neural interconnection between both the homeostatic and non-homeostatic brain pathways that act in concert to control appetite and food intake (figure drawn by author).

Non-homeostatic or hedonic control of food intake is regulated via several neurotransmitters, including dopamine, serotonin (5-HT), opioids, and cannabinoids [28,29,30]. This complex neurobiological circuitry determines the two key valence attributes of food, these being “wanting” and “liking”. The “wanting” of food is primarily determined by the dopaminergic mesolimbic reward pathway (Figure 2); dopamine neurons within the ventral tegmental area (VTA) and the substantia nigra project to the prefrontal cortex, nucleus accumbens (NA), amygdala, and hippocampus. Thus, the dopaminergic system is considered integral to driving the incentive motivation of food that leads to increased appetite, cravings, and excessive calorie consumption. In contrast, there is the hedonic or “liking” aspect of non-homeostatic control, which is known to involve the opioid and cannabinoid pathways.

Furthermore, as previously indicated, there are also strong inter-connections between the homeostatic and non-homeostatic pathways regarding the control of food intake. Indeed, increased hunger caused by an energy deficit is known to intensify cravings. Animal studies have shown that leptin and ghrelin also modulate dopamine reward pathways [31]. The central infusion of ghrelin enhances dopamine release in the NA in response to food cues [32]. In addition, the activation of AGRP neurons using chemogenic technology increases dopamine release in the NA in the presence of food stimuli [33]. Thus, this demonstrates that the key orexigenic hormones (ghrelin) and neuropeptides (AGRP) increase the reward value of food, which in turn is hypothesised to be linked to craving and uncontrolled food patterns (Figure 1).

Glucagon-like peptide 1 (GLP-1) is an incretin hormone released from intestinal cells post-prandially which regulates insulin secretion, gastric motility, and satiety. GLP-1 has a very short half-life in the blood, but long-lasting analogues have been developed and are very effective agents for reducing appetite, body weight, and blood glucose levels. Relevant to weight control, GLP-1 acts in several sites in the brain [34]. Actions in the brainstem nucleus of the solitary tract (NTS) are sufficient to regulate satiety, while actions in the brainstem area postrema (AP) drive nausea [34].

GLP-1 can also act, as mentioned earlier, in the hypothalamus by stimulating the POMC neurons in the arcuate nucleus, to promote satiety, but its influence on hedonic eating likely results from GLP-1 receptors found in the mesolimbic structures like the VTA and NA [35]. These structures interestingly receive input from the NTS that produces GLP-1 centrally and exerts a direct GLP-1 influence on the mesolimbic pathway (Figure 2).

In addition to homeostatic hunger, stress is known to exacerbate uncontrolled eating and to influence the control of food intake. Over 20 years ago, Dallman and colleagues championed the comfort food hypothesis, which stipulates that stress drives the increased consumption of highly palatable foods as a means to control aberrant activation of the hypothalamic–pituitary–adrenal (HPA) axis [36]. In response to stress, neurons within the paraventricular nucleus of the hypothalamus produce corticotropin-releasing factor (CRF) and arginine vasopressin (AVP), which are secreted into the hypophyseal portal circulation to increase adrenocorticotropin (ACTH) secretion from the corticotrope cells of the anterior pituitary gland. In turn, ACTH promotes cortisol secretion from the adrenal gland, which provides negative feedback to the brain and the pituitary gland to ultimately control the HPA axis. However, chronic stress leads to persistently high glucocorticoid concentrations, which increases motivation to consume hyperpalatable foods enriched in dietary fat and sugar. Indeed, during the COVID-19 pandemic, emotional overeating was associated with increased stress-associated intake of high-sugar foods [37]. The increase in the consumption of high-fat and high-sugar foods is considered self-medicating as the increased intake of “comfort food” provides a dampener on the HPA axis to reduce cortisol/glucocorticoid secretion [8,36].

Stress-induced cravings have also been linked to altered mesolimbic reward circuitry. Stress is known to increase the hunger-promoting hormone ghrelin [38] and elevated ghrelin levels can in turn increase the reward salience associated with food [32]. In addition, both cortisol and CRF are also known to provide input to the mesolimbic dopaminergic system [39]. In a world where individuals are experiencing an increase in environmental stressors, the impact of stress on eating and food cravings deserves to be further examined. Furthermore, in the clinical setting, it is fundamental to address the impact of stress and its role in the manifestation of cravings. This review will address ways in which one can reduce stress to combat uncontrollable eating and, importantly, to reduce cravings.

## 4. Identifying Cravings and Their Spectrum of Manifestations

Due to the lack of objective markers, cravings are measured via self-reported questionnaires and rating scales. There are many validated self-reported questionnaires and tools for identifying cravings (for a detailed overview, see [40,41,42]). The review by Taylor [42] also includes a comprehensive overview of the strengths and weaknesses of each measure and a decision tree for selecting the most appropriate questionnaire based on several parameters, including time frame (i.e., past, current, trait) and type of food (i.e., general or specific). Of note, this decision tree can be used to inform researchers and clinicians’ choice of questionnaire. Nevertheless, we provide a detailed overview of commonly used trait/past and state food craving questionnaires alongside their measurement domain and strengths and weaknesses of each measure in Table 1.

There are several highlights worth noting when it comes to medical weight management and the use of anti-obesity medications. Specifically, the Control of Eating Questionnaire (CoEQ) and the Food Cravings Inventory (FCI) have most commonly been used in the assessment of medications with an FDA label for cravings. Tonic experiences of cravings have been assessed consistently using the CoEQ, which is used to not only identify cravings, but also to examine the severity and to distinguish the type of craving, be it sweet or savoury, alongside positive mood. The questions used are straightforward and can be used in clinical consultation as part of history taking; however, the questionnaire does not assess frequency and the strength of craving separately. The FCI is also widely used by clinicians to assess the frequency of cravings for specific types of foods (i.e., high-fat foods, sweet foods, carbohydrate-rich foods, and high-fat fast foods) over the past month.

In research, food cravings are typically measured via the Food Cravings Questionnaires (FCQ), which assesses multi-dimensional aspects of cravings, including cognitive, emotional, behavioural, physiological, and contextual influences. The FCQs include state (S)- and trait (T)-based versions [43]. The state-based and reduced 15-item state version [44] assess momentary food cravings that may be affected by manipulations (e.g., exposure to food cues), while the 39-item trait version assesses the frequency of food cravings. Whilst the latter does not regard momentary fluctuations in food cravings, it does regard shifting during the course of a longer-term weight loss treatment or interventions targeting food cravings [48]. A limitation of the FCQ-T is its length as it can be burdensome for participants to complete. The General Food Craving Questionnaire—Trait (GFCQ-T) and QFCQ—State (GFCQ-T) are modified versions of the FCQ-T and FCQ-S but are used to assess a general desire or urge to eat rather than a desire for specific foods or types of foods [45]. The Craving Experience Questionnaire was designed to assess multi-sensory aspects of both state and past cravings via a 10-item strength version and a 10-item frequency version [47]. Nevertheless, it is a single-dimensional measure as it only assesses the cognitive aspects of craving (e.g., thinking about food) based on EI theory rather than the emotional, behavioural, or physiological aspects, which the FCQs measure. Finally, state cravings can be measured via a single-item rating scale, which assess the current desire or urge to eat a specific food [46]. Single-item rating scales are useful for repeated assessments over a short period as they are brief and simple to complete, but they do not capture the multi-sensory or multi-dimensional nature of food cravings.

To be useful in medical weight management, a scale needs to be simple but more than a screening tool and provide a sufficient depth of measure and characterisation of the phenomenon. Furthermore, it needs to identify individuals that should be assessed for severe forms of uncontrolled eating such as those with binge eating disorder, which can be identified, and the severity of which can be quantified using tools such as the Binge Eating Scale [49].

### New Advances in Craving Measurement

Self-report measures are subject to bias, given the reliance on retrospective accounts of craving intensity or frequency, which can result in under-reporting or recall bias [50]. An alternative method for assessing food cravings is the Ecological Momentary Assessment (EMA), which involves assessing cravings as they occur in daily life using electronic prompting delivered via smartphones. EMA is well placed to assess not only state food cravings, but also behavioural consequences (e.g., regardless of whether food intake occurred) [19,51]. EMA can also be used to assess in-the-moment strategies for mitigating such cravings as well as the preceding mood or motivational states or other external factors that may have generated the cravings. Using EMA methods, the reporting of cravings can be prompted based on a pre-specified researcher/clinician schedule (e.g., morning, noon, evening) or a participant/patient ad hoc basis (e.g., as the cravings occur throughout the day). EMA can be used to deploy standardised craving questionnaires such as the Food Craving Questionnaire—State version [52] or single-item visual analogue scale (VAS) or rating scale [51]. Indeed, single-item scales are the most commonly used approach in the cravings space more broadly (e.g., across substance and behavioural addictions [53]); given their convenience they can effectively capture dynamic changes over time [54], which is further enhanced via the use of EMA techniques.

## 5. Craving Management Options

The management of cravings should be integrated with the modification of lifestyle factors in terms of nutritional balance and sleep quality, as well as the promotion of insights for patients to investigate their patterns of behaviour and the psychological underpinnings of that behaviour, with the aim to improve resilience alongside medication where necessary. The medications that will be discussed are those that are approved for weight management and have evidence for cravings control. They include GLP-1 options such as Semagluutide and Tirzepatide and the combination of bupropion and naltrexone, or Contrave.

### 5.1. Lifestyle Factors

Macronutrient balance has an impact on cravings, with a loading of protein relative to carbohydrate favouring control of cravings. Yang and Tucker [55] showed that consuming a high-protein (30 g) breakfast reduced cravings, as measured by the General Food Cravings Questionnaire, compared to an isocaloric low-protein breakfast (8 g), with the greater carbohydrate content leading to increased cravings. Similarly, Hoertel, et al. [56] showed that a high-protein (35 g) breakfast decreased both sweet and savoury cravings compared with a low-protein (13 g) breakfast and, although less effective than 35 g of protein, was better than skipping breakfast. They also measured homovanillic acid, a peripheral marker of dopamine activity, and found that circulating concentrations correlated with improvements in cravings, suggesting that protein modification reduced cravings via the dopaminergic mesolimbic system. In addition, the effect of protein on craving reduction is prevented by sleep curtailment or sleep disturbances [55,57], suggesting that altered sleep or a lack of sleep contributes to cravings. The latter finding indicates that lifestyle factors should not be managed by clinicians or studied in isolation but should be managed as part of an integrated approach as they are able to modify or impede the progression of other interventions.

Sleep is another important aspect of weight management [58] but, to date, no interventional studies have evaluated the impact of improved sleep alone on cravings. However, concurrent improvements in sweet cravings and sleep are clear and should be part of routine management [59]. If not, there is every likelihood that nutritional change will not make a significant long-term impact on weight and cravings control, as evidenced by the POUND Lost trial (Preventing Overweight Using Novel Dietary Strategies) [60]. This study showed that, not only did those who had sleep disturbance lose less weight on the same dietary intervention, but that weight regain was exacerbated in terms of fat mass per kg weight and that the disinhibition of cravings mediated this change, as measured by the Three Factor Eating Questionnaire. Thus, in regard to lifestyle factors, both dietary as well as additional physiological factors, such as inadequate or poor sleep, influence weight loss success via the modulation of cravings. Furthermore, this highlights the importance of comprehensive lifestyle approaches that not only include dietary modifications, although they are fundamental to the control of cravings and long-term weight loss efficacy.

Exercise is another pillar of lifestyle interventions for weight management. Despite the demonstrated importance for long-term weight loss maintenance, the role of exercise in the initial stages of weight loss is less significant and it does not seem to have a direct impact on cravings control due to the compensatory increase in energy intake [61]. There is a counterintuitive association between uncontrolled eating in terms of binge eating or emotional eating, with the latter not showing improvements [62] or potentially increasing with vigorous intensity exercise in trial settings [63]. Nonetheless, in long-term community-based studies, exercise embedded into behavioural weight loss programmes was shown to mediate long-term impacts on emotional eating over 2 years [64] and is indirectly important when it comes to cravings due to its effects on building psychological resilience and improving mood [65]. The latter is likely related to the dopaminergic pathways outlined earlier [66] and impacts on brain-derived neurotrophic factors (BDNF), which are reviewed elsewhere [67].

### 5.2. Psychological Interventions

Psychological interventions used to reduce cravings have centred around cognitive behavioural therapy (CBT) [68,69] and acceptance-based therapies such as mindfulness-based techniques for eating practises [70,71] and Acceptance and Commitment Therapy (ACT) [72,73]. These techniques are reviewed elsewhere in relation to overall weight management [74].

In terms of cravings, CBT looks beyond the typical lifestyle behavioural imperatives of weight loss, such as caloric restriction and improved physical activity, and delves into the cognitive processes needed to sustain these behavioural changes by removing cognitive obstacles that are likely to impede long-term change [75]. Some of these critical cognitive processes include cognitive flexibility and learning to be adaptable, especially when it comes to dietary guidelines. This enables individuals to see adversity or weight loss relapse to learn and grow and to have a greater appreciation for weight loss maintenance and long-term change as part of a new way of life, rather than the focus being on adherence to strict meal plans and short-term weight loss outcomes. Indeed, CBT has been used to improve treatment outcomes and reduce food cravings in patients who were morbidly obese and undergoing bariatric surgery [76]. CBT also offers an important aspect of managing cravings, namely, to instigate a plan to regularise meals, particularly at the severe end of the uncontrolled eating spectrum; such is the case in binge eating disorder [77]. In brief, learning to move away from a restrictive or deprivation mindset with its subsequent swing to binge episodes is managed by encouraging three planned meals a day with at least two planned snacks. A critical synthesis of the treatment modalities specific for binge eating disorder have been reviewed extensively previously and are not within the scope of this narrative review [78,79].

In relation to mindfulness-based techniques, there are two central components, namely, present moment awareness and decentralisation [74,80]. Present moment awareness is the regulation of attention to what is in the moment. This includes that which can be perceived outwardly as well as of inner experience in terms of body sensations, thoughts, and feelings. Awareness of the present moment is distinct from when attention is absorbed and lost on the conceptualisation of the future or being stuck in memories or events of the past. The second component, decentralisation, involves the experiencing of sensations, thoughts, and feelings as transient and distinct from the constant self or sometimes termed the true self or the perceiver of that which is transient. Ultimately, these two components are interlinked and cycling through these components generates realisation in an individual, which is often described as awareness. This can help to negate stress-related eating at its cause and has also been shown to reduce reward-driven eating, facilitating weight loss [81].

Research has shown that a 7-to-8 week mindfulness intervention reduced cravings, as indexed by the General Food Craving Questionnaire—Trait, relative to a control group in overweight or obese individuals and women experiencing eating-related issues [82,83]. A more recent single-arm study examined a 28-day smartphone-delivered mindful eating intervention, which improved craving-related eating, as assessed via a single-item rating scale administered via ecological momentary experience in women with overweight or obesity [84]. A longer-term single-arm study examining another app-based mindful-eating intervention targeting physiological hunger and satiety perceptions reduced both cravings and body weight over a 12-month period in individuals with obesity [85]. These findings should be confirmed in larger, more rigorous randomised controlled clinical trials and remain to be compared with or used as an adjunct to existing effective management options. Nevertheless, mindfulness-based intervention strategies indicate preliminary effectiveness for reducing food cravings.

A key difference in handling cravings via behavioural strategies is the use of strategies focused on control, namely, modifying versus accepting subjective experiences, including thoughts and emotions, to promote behaviour change. Control-based strategies such as CBT rely on distraction or the suppression of cravings and cognitive re-structuring. In contrast, acceptance-based strategies provoke the acceptance of the craving [82]. Only a few studies have directly compared control- versus acceptance-based strategies for reducing cravings. These studies have shown that acceptance-based strategies are more effective for reducing food cravings in healthy-weight students and women with excess weight, as indexed by the The Food Craving Questionnaire—State and daily single-item reports of craving frequency [52,86,87]. Such acceptance-based strategies focused on ACT, in which participants were taught that cravings are normal, outside of our control, and to accept them as they are over a 2 h programme. Further techniques included ‘defusion’, whereby participants were provided with strategies to step back from cravings [73]. Nevertheless, these studies have been conducted in small samples and have typically lacked a control group. Furthermore, studies did not directly compare mindfulness-based techniques with CBT. Taken together, brief acceptance-based strategies such as ACT appear to be a promising behavioural strategy for reducing food cravings, but long-term term clinical studies are warranted, as are studies comparing mindfulness-based techniques and ACT with control-based approaches such as CBT.

It has been proposed that control-based techniques may be counterproductive, whereby aiming to suppress or change thoughts related to cravings can increase not only the frequency, but intrusiveness of such thoughts [88,89,90]. From a theoretical perspective, this idea aligns with Elaborated Intrusion (EI) theory, such that control-based strategies may enhance elaboration given repeated checking regarding the success of craving suppression attempts [91]; however, this remains to be validated for cravings in the eating domain. As such, novel techniques based on EI theory have emerged that aim to enhance top-down control over craving. These include brief imagery techniques such as body scanning and guided imagery or visualisation, which include components of mindfulness such as relaxation, but do not explicitly focus on thought acceptance and non-judgement. Both techniques were more effective than mind wandering for preventing an increase in food cravings following an overnight fast, as based on a single-item measure and the Craving Experience Questionnaire in a healthy student population [92]. Another intervention targeting the elaboration stage is decentring, a type of mindfulness strategy based on the idea that thoughts and emotions are temporary mental states that can be separated from an individual, which aims to disrupt cravings by diverting attention [93]. Several studies have shown that decentring and visualisation are effective for reducing cravings both in laboratory [94,95] and field settings [96]. While few studies have directly compared the two techniques, there is some evidence to suggest that visualisation is more effective than decentring for reducing craving intensity in self-reported chocolate cravers [97]. Overall, such findings provide further support for the use of brief mindfulness-based strategies for combating food cravings, which has clinical relevance.

Of note, there are many other psychological strategies targeting food cravings that have been tested primarily in laboratory settings. These include top-down strategies such as cognitive regulation (e.g., reappraisal, distraction, suppression, acceptance) and imagery (e.g., functional imagery training, multisensory mental imagery, episodic future thinking [98,99,100,101]) as well as bottom-up strategies such as conditioned cue reactivity (e.g., cue exposure and response prevention), cognitive control training (i.e., attentional bias modification, food-specific inhibitory control training, approach–avoidance training), and biofeedback/neurofeedback training. Whilst it is beyond the scope of the current review to examine each of these strategies in detail, a recent meta-analysis found small, but significant, effects of such techniques on cravings and food intake [102]. The most effective interventions for improving food intake were cue exposure (bottom-up) and reappraisal (top-down), while for reducing food cravings, top-down cognitive regulation strategies (reappraisal, suppression, and distraction), were the most effective. The latter finding contrasts with previous work indicating that acceptance-based techniques were more effective than CBT, but there was a paucity of studies examining acceptance-based techniques included in the meta-analysis. Indeed, mindfulness-based interventions were not included in the meta-analysis. Furthermore, most of the included studies involved healthy samples and single training sessions conducted in laboratory settings. Thus, these techniques should be validated over longer-term periods in clinical populations (e.g., patients with overweight or obesity or binge eating disorder) and conducted in real world settings.

### 5.3. Medications

Anti-obesity medications have been increasingly studied in terms of their capacity to promote satiety and control cravings, but their behavioural and psychological components are not instituted as part of the non-pharmacological components of their management. Nonetheless, detecting changes to cravings in evaluating the efficacy and safety of anti-obesity medications has predominantly featured in the Control of Eating Questionnaire as the predominant modality in which the phenomenon has been affected, with the scientific vigour of a phase 3 double-blind, randomised, controlled clinical trial.

To date, most studies have focused on the effectiveness of a GLP-1 analogue in the control of cravings [103]. Liraglutide is a once-daily injection, and the first GLP-1 analogue introduced for weight management. It is gradually being superseded by more effective and more convenient approaches for use once weekly by GLP-1s like Semaglutide and Tirzepatide (Table 2). When first evaluated for safety and efficacy in phase 3 clinical trials known as the SCALE studies (The Satiety and CLinilca Adiposity–Liraglutide Evidence) in 2015, cravings were not measured in the study population as the understanding of cravings and their significance in weight loss had not gained traction, unlike the more recent phase 3 RCTs for Semaglutide, known as STEP trials, or Tirzepatide, known as the Surmount trials.

In the STEP trials (Semaglutide Treatment Effect in People Living with Obesity), a subset of patients (*n* = 174) were evaluated using the Control of Eating Questionnaire. Once-weekly administration of Semaglutide (2.4 mg) reduced sweet cravings for up to one year and savoury carvings for the entire two-year period. Semaglutide also improved reported difficulties in resisting cravings and reduced overall difficulty in controlling eating for the full two years of the study.

Tirzepatide is a dual GLP-1/GIP, or glucose-inhibitory peptide, agonist approved for the treatment of diabetes and obesity in the USA and more recently in Australia. Its effects on craving were evaluated in a randomised controlled trial (*n* = 27) using a food preference questionnaire and the Food Craving Inventory [104]. This study showed that at 8 and 18 weeks of treatment, Tirzepatide (15 mg) decreased overall food cravings for sweets, carbohydrates, and fast foods, but not for foods high in fat or for fruits and vegetables. Nevertheless, this effect occurred prior to significant weight loss.

Contrave is a combination of naltrexone and bupropion, it is another weight loss medication, and is the best studied of all the weight loss medications for its impact on cravings; of note the data set includes functional MRI data [105] showing changes in brain activity that are consistent with increased executive control. In a study with ~630 patients per arm, Contrave caused significant increases in craving control over 56 weeks, as assessed by the Control of Eating Questionnaire [106,107]. Furthermore, patients with the greatest reductions in cravings showed the biggest weight loss at 56 weeks.

For completeness, other weight management medications such as orlistat have not been assessed for craving control and, although no longer available, the combination of lorcarserin and phentermine has been studied and shown to reduce both weight and cravings, as assessed by the Food Cravings Inventory and Control of Eating Questionnaire [11]. It remains to be seen whether combination therapy with GLP-1 and Contrave will be more efficacious in craving control and provide additional weight loss, as discussed later on.

### 5.4. Integrating Medications with Lifestyle and Psychological Strategies

Herein, it is proposed that medical weight management requires the successful use of medication, whereas cravings control involves integrating lifestyle and psychological approaches, as outlined above. An example of a holistic person-centred approach for diabetes management was recently published in a consensus statement by the American Diabetes Association and the European Association for the Study of Diabetes in 2022 [108]. To date, there is no similar publication that the authors are aware of for medical weight management, let alone for cravings control. The same can be argued to be a requirement for medical weight management of cravings control as this integration would not only likely improve the efficacy of medical weight management but also reduce the likelihood of medication side effects.

Nausea, for example, is common side effect with GLP-1 analogues (Table 2) due to the slowing of gastric emptying [109] as well as through a direct impact in the area postrema in the brainstem [110]. To reduce nausea, it is widely recommended that having small regular meals, in keeping with the principles of managing uncontrolled eating, is encouraged along with the slowing down of eating, in keeping with the mindful eating strategies of appreciating the taste and being sensitive to internal cues of fullness. By doing so, the patient can stop eating once nearing satiation, a sensation that is unexpectedly early in the meal when a GLP-1 analogue is initiated, often leading to the experience of discomfort only too late, sometimes progressing to vomiting.

Furthermore, the efficacy of weight loss medication can also improve when lifestyle measures are implemented concurrently, as seen in intensive behavioural modification strategies. The phase 3 RCT for Contrave, known as the COR trial, had an intensive behavioural therapy (IBT) trial that enhanced the effect of weight loss achieved from 7% to 11% [111]. IBT, however, is not universal in enhancing weight loss effects when it comes to stronger weight loss medications in the short term, as observed in high-dose Semaglutide 2.4 mg (Wegovy) in the STEP trials, as the weight loss achieved was not dissimilar without IBT [112]. It should be noted that the IBT in the STEP trial did not address the cognitive aspects of CBT for cravings control, let alone mindfulness-based eating. The eating recommendation emphasised hypocaloric dieting rather than what is proposed here, namely macronutrient balance, the regularity of meals, and the enjoyment of food.

This proposal is important as the study of the data of the STEP trials shows that close to 15% of individuals on 2.4 mg Semaglutide and close to 5% of individuals on GLP-1 Analogue Tirzepatide at 15 mg in the Surmount trials did not achieve more than 5% weight loss; it would be important for future studies to see if these individuals are those that have a problem with hedonic eating or craving control and whether strategies to tackle cravings can be targeted at them. To date, the aforementioned psychological therapies have not been studied in combination with medication therapy and this is a deficiency that urgently warrants addressing.

The integration of medication therapy with lifestyle and psychological modalities requires a treating physician who appreciates the role and timing of these interventions through the patient’s journey. In effect, the role of the treating physician in medical weight management is not just to prescribe anti-obesity medication, considering the risks versus benefits, but to identify individuals that need more than just hypocaloric diets and exercise, asking questions with a non-judgemental approach in order for that which can be a difficult or shameful behaviour for patients to admit to be openly and honestly discussed [113]. This identification starts with normalising food cravings as a behaviour, which is based on our understanding of the pathophysiology, and having an initial exploration and assessment with questions that are non-confronting. Once identified, a more formal questionnaire can be used, as mentioned in Section 4 of this review. Table 3 outlines some common questions that can be asked of patients in a non-judgemental way to help explore cravings as a phenomenon.

**Figure 3:**
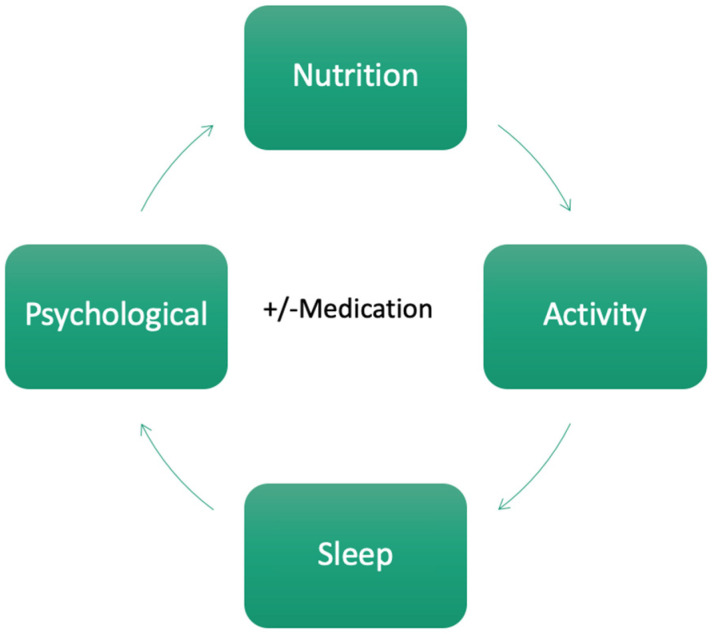
A proposed iterative approach to medical weight management including lifestyle factors (i.e., nutrition, activity, and sleep) as well as psychological interventions, alongside the potential use of medications (figure drawn by author).

The treating physician is not alone in the implementation of a holistic approach for cravings control. The availability of a multidisciplinary team (MDT) is paramount in this process. The MDT would usually involve dietitians, psychologists, exercise physiologists, and sleep physicians, but also less traditional health professions such as health coaches, personal trainers, yoga teachers, and counsellors. Nevertheless, having an MDT does not necessarily mean that the overall management will be holistic if each individual discipline acts independently and is unaware of the complex interactions between the physical and psychological state of their client. The input from other members of the MDT should be coordinated, communicated, and facilitated by the treating physician to ensure the ongoing improvement of nutrition, sleep, psychological wellbeing, and physical activity, progressing in an iterative fashion with increasing intensity and precision, this constituting the person-centred approach (Figure 3).

### 5.5. New Advances in Craving Management

Future research should investigate emerging treatment strategies to use as an adjunct to existing effective management options or as standalone. Emerging strategies include non-invasive neurostimulation techniques such as repetitive transcranial magnetic stimulation (rTMS) or transcranial direct current stimulation (tDCS) of the prefrontal cortex. A recent meta-analysis found that tDCS successfully reduces cravings, as indexed by trait- (Food Cravings Inventory) and state-based measures of food cravings (the Food Craving Questionnaire—State) [114], albeit most studies were conducted in small samples of healthy participants with frequent food cravings or individuals with obesity. However, larger trials examining impacts on food consumption and other clinical outcomes are needed in patients with obesity before the use of tCDS as a management option is warranted.

## 6. Future Directions

There is a strong need for research into the management of cravings induced by psychotropic medications, particularly with the rapid rise of incretin hormone-based therapies [115]. The development of guidelines for managing the metabolic sequelae of psychotropic medication is paramount given the growing mental health crisis. Furthermore, the use of combination medication therapy with an incretin base should receive attention, given the unlikely collaboration between different pharmaceutical companies with the increasing use of GLP-1 analogues with Contrave or Phentermine. Finally, future craving management options should involve an integrative approach, as outlined in Figure 3, and be studied, especially in those who receive medications but are insufficiently responsive. The challenge is to bring traditional disparate fields of study that span the physiological and psychological into both the study and the clinic.

## 7. Conclusions

Long-term weight loss needs to consider the phenomenon of cravings and its many manifestations of uncontrolled eating. There are several ways the phenomenon can be identified, but non-judgemental history taking and the normalisation of the behaviour is the first step, along with the potential use of questionnaires to follow up and identify the progression of the phenomenon. The options for management range from lifestyle interventions including macronutrient manipulation, sleep quality improvement, mindfulness-based psychological therapies, and CBT, along with a select group of anti-obesity medications including Semaglutide, Tirzepatide, and Contrave. A holistic approach that integrates these management options is recommended for patients experiencing food cravings to improve behavioural and clinical outcomes. The changes made should be implemented in an iterative fashion as the factors affect each other which, by doing so, would allow for intensification and a precise approach, changing both from patient to patient and across a patient’s lifespan.

## Figures and Tables

**Table 1:** Summation of domains, strengths, and weaknesses of questionnaires commonly used to assess state and trait food cravings.

Questionnaire	Craving Domain	Strengths	Weaknesses
Past/Trait Food Cravings			
Control of Eating Questionnaire (CoEQ)	Tonic experience, frequency, severity, type of craving (sweet, savoury), positive mood	Strong criterion validity, assesses hunger/satiety; four-factor structure replicated	Frequency and severity are not measured separately
The Food Craving Inventory (FCI)	Frequency of specific food cravings across four types of food (high fat, sweets, carbohydrates, and fast-food fats)	Four-factor structure replicated in clinical samples; suitable for cross-cultural use; good test–retest reliability and criterion validity; useful for clinicians	Mixed evidence for predictive validity
Food Craving Questionnaire—Trait (FCQ-T) [43] and FCQ-T-reduced (FCQ-T-r) [44]	Multi-dimensional (cognitive, emotional, contextual, behavioural, physiological); commonly craved foods	FCQ-T: good test–retest reliability for total score; psychometric evaluation in clinical samples; good predictive, construct, and criterion validity; suitable for cross-cultural use. FCQ-T-r: shorter/less burdensome (15 items; single-factor structure replicated (healthy/clinical samples))	FCQ-T: lengthy/burdensome for participants to complete (39 items); risk of inaccurate responding or missing date; lower test–retest reliability in overweight and obesity relative to healthy samples; unstable factor structure
General Food Craving Questionnaire—Trait (GFCQ-T) ^1^ [45]	Multi-dimensional (cognitive, emotional, contextual, behavioural, physiological); general desire for food or to eat	Good test–retest reliability for total score; evidence of criterion validity; briefer than FCQ-T; suitable for cross-cultural use	Factor structure not replicated
State Food Cravings			
Single-item craving rating scale [46]	Desire or urge to eat	Brief and simple	Single-dimensional (cognitive aspect)
The Craving Experience Questionnaire (CEQ) [47]	Strength (state) and frequency (trait) of craving-related thoughts and urges, vividness of imagery, intrusiveness of thoughts	Researcher-defined timeframe (past cravings); specific foods or food generally; brief (10 items); theoretical basis (EI theory); multi-sensory aspect of cravings; strength (state) and frequency (past) of cravings assessed separately	Single-dimensional (cognitive aspect); criterion validity not assessed
Food Craving Questionnaire—State (FCQ-S) [43]	Multi-dimensional (cognitive, emotional, contextual, behavioural, physiological); commonly craved foods	Sensitive to acute fluctuations; brief (15 items); specific-food; strong construct validity; tested in clinical samples; suitable for cross-cultural use	Low test–retest reliability; poor predictor of long-term weight change; lack of sensitivity detecting differences between individuals with or without weight-related issues
General Food Craving Questionnaire—State (GCFQ-S) ^2^ [45]	Multi-dimensional (cognitive, emotional, contextual, behavioural, physiological); general desire for food or to eat	Sensitive to acute fluctuations; brief (15 items); tasty food in general; strong construct and criterion validity	Low test–retest reliability

^1^ A modified version of the FCQ-T; ^2^ a modified version of the FCQ-S.

**Table 2:** Common anti-obesity medications and their expected average weight loss along with cravings reduction data and common side effects.

Medication	Average Weight Loss	Cravings Reduction	Common Side Effects (>5% and >2 × Placebo)
Liraglutide (Saxenda)	8–10% in 1 year RCT (SCALE Trials)	Yes (mild effect on Food Cravings Inventory)	Nausea and vomiting Diarrhoea Constipation Dyspepsia
Semaglutide (Wegovy)	15–17% in 1 year RCT (STEP Trials)	Yes (moderate effect on Control of Eating Questionnaire)	Nausea and vomiting Diarrhoea Constipation Dyspepsia
Tirzepatide (Mounjaro)	22–24% in 1 year RCT (Surmount Trials)	Yes (moderate effect on Control of Eating Questionnaire)	Nausea and vomiting Diarrhoea Constipation Dyspepsia
Naltrexone-Bupropion (Contrave)	8–11% in 1 year RCT (COR Trials)	Yes (moderate effect on Control of Eating Questionnaire)	Nausea and vomiting Constipation Dizziness Hot flushes Dry mouth
Orlistat (Xenical)	3% in 6 months RCT	Unlikely	Diarrhoea Faecal urgency Abdominal discomfort
Phentermine (Duromine)	5–7% in 6 months RCT	Yes (Mild effect on Trait and State Food Cravings Questionnaire)	Dry mouth Restlessness

Note: SCALE—The Satiety and Clinical Adiposity—Liraglutide Evidence; STEP—Semaglutide Treatment Effect in People with obesity.

**Table 3:** Common questions used to start the conversation around cravings.

Question About Cravings
After a long day, I can crave carbs. Does that happen to you?
2. Is that craving usually for sweet or savoury or both?
3. When do cravings normally happen? During the day, late afternoon, or late at night?
4. Do you feel judged by others or even yourself when you are eating to satisfy cravings?
5. Do you find satisfying cravings lead to overeating to the point of discomfort or distress? (If yes, consider further exploration using Binge Eating Disorder Scale, see Section 3)

## Data Availability

No new data were created or analysed in this study. Data sharing is not applicable to this article.
